# Correction: Guo et al. Optimization Mechanism of Nozzle Parameters and Characterization of Nanofibers in Centrifugal Spinning. *Nanomaterials* 2023, *13*, 3057

**DOI:** 10.3390/nano16060381

**Published:** 2026-03-23

**Authors:** Qinghua Guo, Peiyan Ye, Zhiming Zhang, Qiao Xu

**Affiliations:** 1 Hubei Digital Textile Equipment Key Laboratory, Wuhan Textile University, Wuhan 430200, China; 2115373039@mail.wtu.edu.cn (Q.G.); 2115373044@mail.wtu.edu.cn (P.Y.); 2 School of Mechanical Engineering and Automation, Wuhan Textile University, Wuhan 430200, China; 2006109@wtu.edu.cn

In the original publication [1], reference [5] has been retracted prior to the publication. Concerns were raised, and the authors want to replace it with the following reference:

“5. Ahmed, J.; Gultekinoglu, M.; Edirisinghe, M. Recent developments in the use of centrifugal spinning and pressurized gyration for biomedical applications. *WIREs Nanomed. Nanobiotechnol.* **2024**, *16*, e1916”

The original sentence citing this reference has been revised to the following sentence:

“Centrifugal spinning technology produces nanofibers with unique characteristics, such as high surface-to-volume ratio, tunable porosity, and distinct morphological properties [5].”

The authors state that the scientific conclusions are unaffected. This correction was approved by the Academic Editor. The original publication has also been updated.
